# Improving models of fine root carbon stocks and fluxes in European forests

**DOI:** 10.1111/1365-2745.13328

**Published:** 2020-01-10

**Authors:** Mathias Neumann, Douglas L. Godbold, Yasuhiro Hirano, Leena Finér

**Affiliations:** ^1^ Institute of Silviculture University of Natural Resources and Life Sciences Vienna Austria; ^2^ Institute of Forest Ecology University of Natural Resources and Life Sciences Vienna Austria; ^3^ Global Change Research Centre Academy of Sciences of the Czech Republic Prague Czech Republic; ^4^ Graduate School of Environmental Studies Nagoya University Nagoya Japan; ^5^ Natural Resources Institute Finland Joensuu Finland

**Keywords:** below‐ground, coring, decomposition, ingrowth, minirhizotron, necromass, rhizosphere, soil

## Abstract

1. Fine roots and above‐ground litterfall play a pivotal role in carbon dynamics in forests. Nonetheless, direct estimation of stocks of fine roots remains methodologically challenging. Models are thus widely used to estimate these stocks and help elucidate drivers of fine root growth and turnover, at a range of scales.
2. We updated a database of fine root biomass, necromass and production derived from 454 plots across European forests. We then compared fine root biomass and production to estimates obtained from 19 different models. Typical input variables used for the models included climate, net primary production, foliage and above‐ground biomass, leaf area index (LAI), latitude and/or land cover type. We tested whether performance could be improved by fitting new multiple regression models, and explored effects of species composition and sampling method on estimated fine root biomass.
3. Average fine root biomass was 332 g/m^2^, and necromass 379 g/m^2^, for European forests where the average fine root production was 250 g m^−2^ year^−1^. Carbon fraction in fine roots averaged 48.4%, and was 1.5% greater in broadleaved species than conifers.
4. Available models were poor predictors of fine root biomass and production. The best performing models assumed proportionality between above‐ and below‐ground compartments, and used remotely sensed LAI or foliage biomass as key inputs. Model performance was improved by use of multiple regressions, which revealed consistently greater biomass and production in stands dominated by broadleaved species as well as in mixed stands even after accounting for climatic differences.
5. *Synthesis.* We assessed the potential of existing models to estimate fine root biomass and production in European forests. We show that recalibration reduces by about 40% errors in estimates currently produced by the best available models, and increases three‐fold explained variation. Our results underline the quantitative significance of fine roots (live and dead) to the global carbon cycle.

## Graphical abstract

Fine roots are essential for plant growth and represent a substantial carbon stock and large share of primary production of forests. Existing models for fine root biomass and production performed poorly validated with a new database on European forests. Recalibration of multiple regression models increased the coefficient of determination up to threefold. We also explore options to improve fine root estimates further by considering necromass or rooting depth.

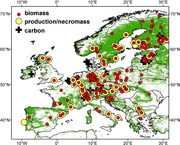

## 1 INTRODUCTION

In forests, soils are the dominant carbon storage pool (Lal, 2005; Pan et al., 2011). While fine roots are a relatively small contributor to below‐ground C at any point in time (Jackson, Mooney, & Schulze, 1997; Liski, Perruchoud, & Karjalainen, 2002), their fast turnover ensures they provide a major contribution to soil organic carbon (SOC) (Bray & Gorham, 1964; Gill & Jackson, 2000). Fine root production—the flux of carbon into the SOC pool—is variously estimated to be between 22% and 36% of net primary production (NPP) at the forest scale (Jackson et al., 1997; Malhi, Doughty, & Galbraith, 2011; McCormack et al., 2015; Yuste et al., 2005). Despite this obvious importance, studies of fine roots remain seriously under‐represented in forest literature, most likely due to their labour‐intensive and time‐demanding nature.

Estimates of fine root biomass and turnover (production) obtained from direct measurements also differ according to the method (Brunner et al., 2013). These methods include soil cores, monoliths and trenches for biomass estimation, and sequential coring, minirhizotrons, and in‐growth cores and nets or bags for production estimates (Addo‐Danso, Prescott, & Smith, 2016). Estimates of biomass are less affected by method than those of production (Addo‐Danso et al., 2016; Brunner et al., 2013; Finér, Ohashi, Noguchi, & Hirano, 2011b; Hertel & Leuschner, 2002). Consequently, there have been calls for standardized approaches (Brunner et al., 2013) and for method comparisons at specific sites (Hertel & Leuschner, 2002; Železnik et al., 2018).

Further difficulties are introduced by the high costs and disturbances associated with the sampling of deep soil layers (Finér, Ohashi, Noguchi, & Hirano, 2011a; Schenk & Jackson, 2002), that mostly lead to underestimation of fine root biomass. For example, in temperate forests the average sampling depth used (47 cm) recovered only 58% of the expected fine root biomass in the entire rooting depth (Finér et al., 2011a). Fine root biomass is thus often derived by extrapolation of root density in surface soils to the so‐called ‘maximum rooting depth’ using knowledge of vertical fine root distribution (Gale & Grigal, 1987; Jackson et al., 1996). Most of these extrapolations are not site or species‐specific and direct measurement (via excavation, cores, etc.) remains the best method for capturing local conditions. Such estimates are still comparatively rare (Finér et al., 2011b; Iversen et al., 2017; Yuan & Chen, 2010).

Limited availability of reliable data, and the challenges of direct sampling, makes attractive the use of models to estimate fine root biomass and turnover. Without modelling of fine root dynamics, scientists would have to exclude below‐ground processes from their ecosystem analyses or limit the work to the few studies which have quantified all components of the C cycle (Gower et al., 2001). A large number of generalized models can be used to estimate fine root biomass and production in forests stands. These models use a range of input parameters. For example, regression models make use of meta‐information of forest stands such as climate, latitude or altitude (Liu et al., 2004; Yuan, Shi, Jiao, & Han, 2018), tree stem diameter (Chen, Chen, Price, & Cihlar, 2002), or above‐ground tree biomass (Härkönen, Lehtonen, Eerikäinen, Peltoniemi, & Mäkelä, 2011; Liski et al., 2002). In contrast, mechanistic biogeochemical‐based approaches assume that fine root turnover is a constant fraction of NPP (Malhi et al., 2011) or utilize carbon allocation patterns and turnover rates (Pietsch, Hasenauer, & Thornton, 2005; Running & Zhao, 2015; White, Thornton, Running, & Nemani, 2000). One of the oldest modelling concepts assumes that the turnover of fine roots is proportional to that of the above‐ground compartments (Chen, Hobbie, Reich, Yang, & Robinson, 2018; Nadelhoffer & Raich, 1992; Raich & Nadelhoffer, 1989; Shinozaki, Yoda, Hozumi, & Kira, 1964).

While forest structure information is rarely used as model inputs (Härkönen et al., 2011; Liski et al., 2002; Malhi et al., 2011; Pietsch et al., 2005), recent studies have shown that competition (e.g. as measured by the mass of foliage of neighbouring trees) helps explain spatial variation in above‐ground litterfall (Neumann et al., 2018), as well as fine root biomass and production (Finér et al., 2011a,b). The availability of remotely sensed forest information from satellites (Friedl et al., 2010; Yan et al., 2016; Yang et al., 2006) or inventory‐based gridded forest structure data (Moreno, Neumann, & Hasenauer, 2017) may also permit large scale and spatially continuous applications. By analogy with above‐ground turnover via litterfall, where climatic variables are often strong predictors (e.g. Adams & Attiwill, 1986; Neumann et al., 2018), fine root turnover also seems likely to be climate‐related.

Candidate models for estimating fine root biomass and production can be tested using validation analysis (Willmott & Matsuura, 2006). Inclusion of other drivers could also improve the applicability of these fine root biomass and production models. (a) Soil chemical properties are strong candidates (Godbold, Fritz, Jentschke, Meesenburg, & Rademacher, 2003; Richter, Hajdas, Frossard, & Brunner, 2013; Yuan & Chen, 2012) and their inclusion is warranted by increased availability of spatially explicit soil information (Fan, Li, & Miguez‐Macho, 2013; Fan, Miguez‐Macho, Jobbágy, Jackson, & Otero‐Casal, 2017). (b) Dead fine roots (necromass) have been shown to be significantly related to fine root biomass and production (Leuschner & Hertel, 2003; Wang et al., 2018). (c) Carbon fractions are now known to vary with species and root diameter (Jackson et al., 1997; Thomas & Martin, 2012). (d) Species composition plays a major role in fine root biomass and production (Finér et al., 2017).

Reviews of fine root biomass, necromass and production are dominated by observations from North America, Europe or China (e.g. Finér et al., 2011a,b; Wang et al., 2018; Yuan & Chen, 2010). Sample distributions nonetheless often show a regional bias, which can mask or exclude important inputs. Bias towards a particular set of climatic conditions could, for example, obscure its role in both biomass and production (e.g. Meier & Leuschner, 2008).

In this study, we focused on continental Europe. We used an updated database of fine root biomass, necromass, production, and carbon fraction for European forests. We compared fine root biomass and production data to the outputs of 19 published models. The continuous updating and complementation of existing fine root databases providing estimates of the contribution of fine roots to carbon stocks, is essential for accurate reporting as required, for example, by greenhouse inventories for Land Use, Land‐Use Change, and Forestry (LULUCF) sector or the Intergovernmental Panel on Climate Change (IPCC). More broadly such databases can be used for improving our understanding of the role of fine roots in global C cycling (Hendricks, Nadelhoffer, & Aber, 1993; Likens, 2013). Our objectives were as follows: (a) to evaluate existing fine root biomass, necromass and production models; (b) to improve models by recalibration or in‐/excluding input variables; and (c) to evaluate drivers of fine root stocks and fluxes, including climate, tree species composition and the sampling method.

## 2 MATERIALS AND METHODS

### 2.1 Fine root database

**Figure 1:**
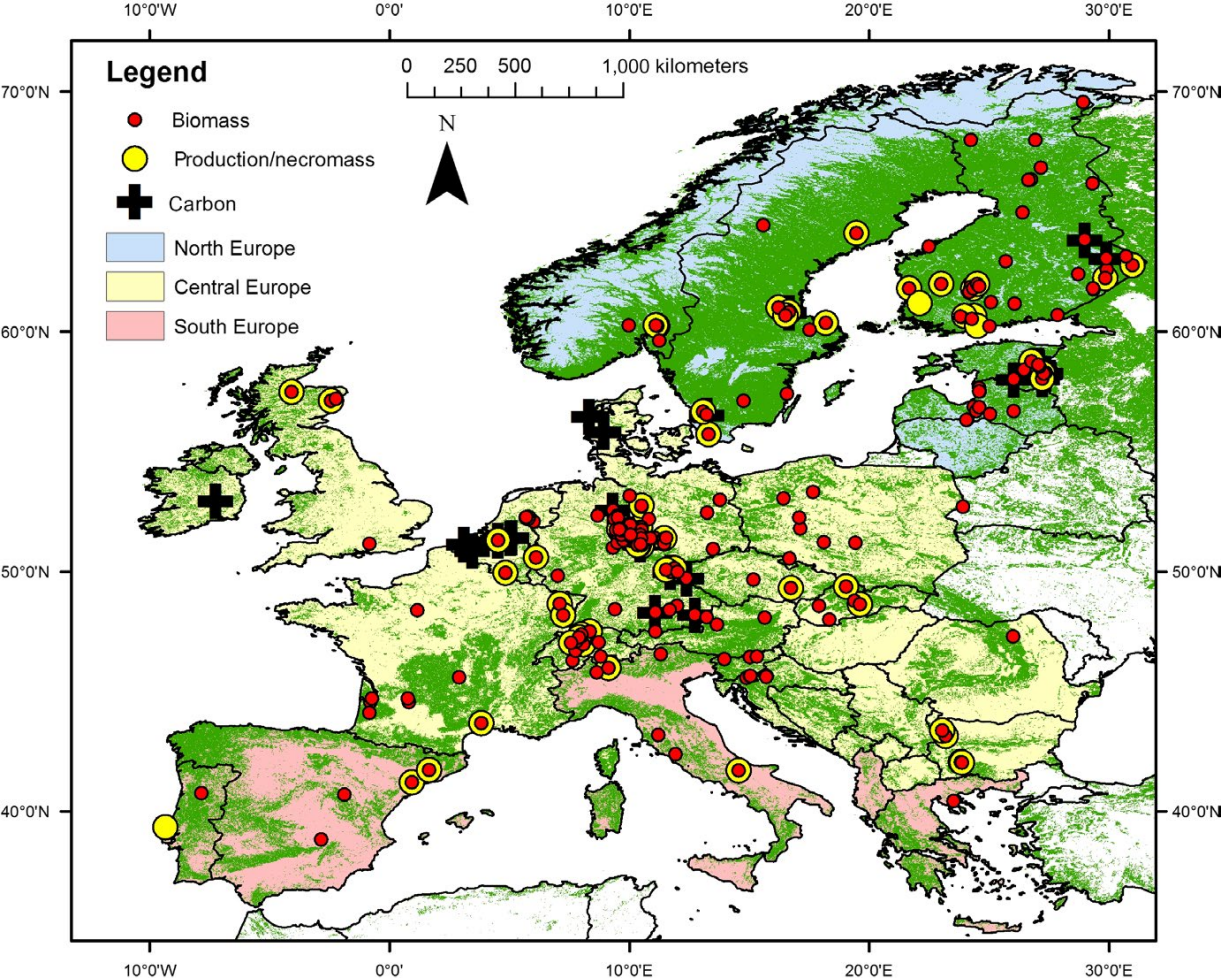
Locations of the forests sites with available fine root data (biomass, necromass, production and carbon fraction) across the three bioregions. Green shaded pixels are forested land cover types of satellite‐derived global MODerate resolution Imaging Spectroradiometer Land Cover (Friedl et al., 2010) [Colour figure can be viewed at http://wileyonlinelibrary.com]

We used the European data from the databases compiled by Finér et al. (2011a,b) and the FRED initiative (Iversen et al., 2017). We complemented the databases using recent publications (see Data sources section for a complete list of references). We only used in situ data on fine roots (diameter <2 mm) of trees and the understorey vegetation, excluding pot experiments and agricultural short‐rotation plantations. We also excluded fine root biomass data obtained by using ingrowth cores or nets. We added information on tree species composition, fine root necromass and carbon fraction, if available. Missing meta‐information such as location or stand structure was added based on other publications for the same sites. We assigned our observations to three bioregions, North, Central and South Europe, which largely correspond to the forest vegetation zones boreal, temperate and Mediterranean (Figure 1).

### 2.2 Fine root biomass models

We used both fine root biomass and production models. There were no models available for estimating fine root necromass. We compiled nine different models (labelled B1 to B9 plus a name tag), which we used for estimating fine root biomass (g/m^2^) for the sites in the database. We then compared the model outputs to the observed values. The input variable of models B1–B3 is remotely sensed leaf area index (LAI, explained in more detail in Section 2.4). The models B1–B3, similar to biogeochemical models (Pietsch et al., 2005; White et al., 2000), derive fine root biomass by converting LAI with carbon fraction, specific leaf area (SLA) and fine root‐to‐leaf mass ratio (FR:LEAF). We obtained these conversion parameters from three widely used sources, to determine the importance of model coefficients independent from the data input. For model B1 (‘LAI MOD17’), we used SLA and FR:LEAF from the MOD17 algorithm providing global NPP using vegetation information derived from the satellite‐mounted sensor MODIS (MODerate resolution Imaging Spectroradiometer) and climate data (Running & Zhao, 2015). SLA and FR:LEAF of models B2 and B3 we took from the parametrizations of Biome BGC, a mechanistic biogeochemical ecosystem model (for North America, model B2, ‘LAI White’, and for Central Europe, model B3, ‘LAI Pietsch’, Pietsch et al., 2005; White et al., 2000). SLA and FR:LEAF from models B1–B3 originate from MOD17 calibration and literature reviews (Olson, Johnson, Zheng, & Scurlock, 2001; Pietsch et al., 2005; Scurlock & Olson, 2002; White et al., 2000). We were not aware of available Biome BGC reparametrizations for boreal or Mediterranean forests in Europe, thus we used one consistent continental parameter set. We provide SLA (m^2^/g C) and FR:LEAF (unitless) in Table S1. Subscript ‘M’ indicates parameters from MOD17 (Running & Zhao, 2015), subscript ‘W’ from White et al. (2000) and ‘P’ from Pietsch et al. (2005). The unit of SLA requires dividing the results of B1–B3 with carbon fraction, i.e. the carbon in fine roots per dry mass. For Equations (B1), (B2), (B3), we assumed carbon fraction (g C/g) to be 0.488 according to Jackson et al. (1997). LAI has the unit m^2^/m^2^.

(B1) $$\text{Fine root biomass}=\text{LAI}/\text{carbon fraction}/{\text{SLA}}_{M}\times {\text{FR:LEAF}}_{M}$$

(B2) $$\text{Fine root biomass}=\text{LAI}/\text{carbon fraction}/{\text{SLA}}_{W}\times {\text{FR:LEAF}}_{W}$$

(B3) $$\text{Fine root biomass}=\text{LAI}/\text{carbon fraction}/{\text{SLA}}_{P}\times {\text{FR:LEAF}}_{P}$$

From several models using annual average temperature, precipitation sum and/or latitude for *Picea abies* [L.] Karst in Europe (Yuan et al., 2018), we evaluated only the best performing model using latitude (˚) as the explaining variable (model B4, ‘Yuan’).

(B4) Fine root biomass=-0.217×Latitude+17.566

Stem biomass is the input variable of model B5 and foliage biomass for models B6–B9. Liski et al. (2002) in a European study assumed that fine root biomass is proportional to stem biomass (g/m^2^) (Eq. B5, ‘Liski’). Härkönen et al. (2011) in a Finnish study instead used foliage biomass (g/m^2^) and published species‐specific fine root–foliage mass ratios (FR:LEAF_H_ for conifers 0.3 and for broadleaves 1.5, corresponding to average site conditions; Eq. B6, ‘Harkonen’).

(B5) Fine root biomass=Stem biomass×0.02

(B6) $$\text{Fine root biomass}=\text{foliage biomass}\times {\text{FR:LEAF}}_{H}$$

Missing information on foliage or stem biomass were amended using reported forest structure information (diameter, stem number, species type) and allometric biomass functions used in Neumann et al. (2018).

Finally, we estimated fine root biomass solely using foliage biomass, which implies that fine root biomass is proportional to foliage biomass (model B7, ‘Foliage biomass’) sensu Shinozaki et al. (1964) and Chen et al. (2018). We also combined foliage biomass with the FR:LEAF ratio provided by Pietsch et al. (2005) and White et al. (2000) and used for models P3 and P4 (model B8, ‘Foliage White’ and model B9, ‘Foliage Pietsch’).

(B7) Fine root biomass=foliage biomass

(B8) $$\text{Fine root biomass}=\text{foliage biomass}\times {\text{FR:LEAF}}_{W}$$

(B9) $$\text{Fine root biomass}=\text{foliage biomass}\times {\text{FR:LEAF}}_{P}$$

### 2.3 Fine root production models

We also compared the outputs of ten fine root production (g m^−2^ year^−1^) models to the observed production values (labelled P1 to P10). The model P1 after Malhi et al. (2011) developed with data from tropical forests assumes that fine root production is proportional to NPP of the forest stand. NPP has the unit g C m^−2^ year^−1^ and for comparison with fine root production from our database has to be converted with the carbon fraction (model P1). For Equations (P1), (P2), (P3), (P4), we assumed the carbon fraction to be 0.488 according to Jackson et al. (1997).

(P1) Fine root production=NPP×0.27/carbon fraction

Analogous to the fine root biomass models, we also tested fine root production models using LAI derived from remote sensing. Models P2 to P4 correspond to models B1 to B3, respectively, with turnover (year^−1^), which is the annual turnover rate of fine roots provided in Table S1.

(P2) $$\text{Fine root production}=\text{LAI}/\text{carbon fraction}/{\text{SLA}}_{M}\times {\text{FR:LEAF}}_{M}\times {\text{turnover}}_{M}$$

(P3) $$\text{Fine root production}=\text{LAI}/\text{carbon fraction}/{\text{SLA}}_{W}\times {\text{FR:LEAF}}_{W}\times {\text{turnover}}_{W}$$

(P4) $$\text{Fine root production}=\text{LAI}/\text{carbon fraction}/{\text{SLA}}_{P}\times {\text{FR:LEAF}}_{P}\times {\text{turnover}}_{P}$$

Liu et al. (2004) provided several regression functions for the estimation of above‐ground total or foliage litterfall using observations from across Eurasia, which were recently validated with observations in Europe (Neumann et al., 2018). By assuming that above‐ground turnover is proportional to below‐ground turnover (Nadelhoffer & Raich, 1992; Raich & Nadelhoffer, 1989) also permitted us to test the models of Liu et al. (2004) in this study. We used in total four models, two developed for total litterfall (model P5, ‘Liu total T + P’ and model P6, ‘Liu total T’) and two for foliage litterfall (model P7, ‘Liu leaf T + P’ and model P8, ‘Liu leaf T’) to avoid presumptions. Equations P5 and P7 use both mean annual air temperature (T; °C) and annual precipitation sum (P; mm) as input variables, while models P6 and P8 require T alone. Models P5 to P8 were applied with downscaled European climate data as input (Moreno & Hasenauer, 2016). In addition, we computed Equations P7 and P8 with a second climate dataset, the WorldClim (WC) data (Hijmans, Cameron, Parra, Jones, & Jarvis, 2005) as input to obtain two additional model outputs, called P9 (‘Liu leaf T + P WC’) and P10 (‘Liu leaf T WC’), which are not shown here as their form is identical with Equations P7 and P8 respectively. WorldClim provides only long‐term averages globally (Hijmans et al., 2005), while the downscaled European climate data have a daily resolution (Moreno & Hasenauer, 2016) and were used for computing the European NPP dataset used in this study (Neumann, Moreno, Thurnher, et al., 2016).

(P5) $$\text{Fine root production}=\exp\left(2.296+0.741\times \log\left(T+10\right)+0.214\times \log\left(P\right)\right)$$

(P6) $$\text{Fine root production}=\exp\left(3.12+0.962\times \log\left(T+10\right)\right)$$

(P7) $$\text{Fine root production}=\exp\left(2.241+0.65\times \log\left(T+10\right)+0.211\times \log\left(P\right)\right)$$

(P8) $$\text{Fine root production}=\exp\left(3.102+0.853\times \log\left(T+10\right)\right)$$

We compared all model predictions versus observations by calculating root‐mean‐square error (RMSE), mean absolute error (MAE), mean bias error and coefficient of determination as described in Willmott and Matsuura (2006). We evaluated the model outputs further by plotting the model residuals against selected gradients (latitude, elevation, stand age and stand density index).

### 2.4 Input data required for the models

We calculated fine root biomass and fine root production with the compiled models (B1–B9, P1–P10) for the sites in our database. The input data of the models was consistent for entire Europe to avoid data gaps and artefacts due to different methodologies. The source for NPP was Neumann, Moreno, Thurnher, et al. (2016), for LAI (Yang et al., 2006) and for temperature (T) and precipitation (P) (Hijmans et al., 2005; Moreno & Hasenauer, 2016). We used periodic annual average NPP, maximum annual LAI, average annual daily temperature and annual precipitation sum from 2000 to 2012 (1960 to 1990 for Hijmans et al., 2005). NPP and LAI data were not available for the period before 2000. Land cover information needed for the land cover‐specific LAI‐based models (Equations (B1), (B2), (B3), (P2), (P3), (P4)) was taken from MODIS Land Cover representing conditions in year 2000 (Friedl et al., 2010) used also in Figure 1.

### 2.5 Accounting for differences in sampling depth

The sampling depth in the field studies was usually much lower than the maximum rooting depth (Jackson et al., 1997; Schenk & Jackson, 2002). Therefore, we extrapolated fine root biomass and fine root production for the entire rooting depth using the concept introduced by Gale and Grigal (1987) and Jackson et al. (1997) according to Equation 1 and compared the extrapolated values to the model outputs (Equations (B1), (B2), (B3), (B4), (B5), (B6), (B7), (B8), (B9), (P1), (P2), (P3), (P4), (P5), (P6), (P7), (P8), P9–P10).

(1) $$\text{extra}=\text{obs}/(1-\beta^{\text{depth}})$$

In Equation 1, obs is the observed fine root data and extra is the fine root data extrapolated to the entire rooting depth. Depth is the sampling depth (cm) reported in the reference. Here *β* is the coefficient determining the shape of the rooting profile after Jackson et al. (1997) with a *β* of 0.943 for Northern Europe (boreal forest), *β* 0.98 for Central European conifers (temperate coniferous forest), *β* 0.967 for Central European broadleaves (temperate deciduous forest) and *β* 0.95 for Southern Europe (sclerophyllous shrubs and trees).

### 2.6 Evaluation of drivers for fine roots by model recalibration

We fitted multiple linear models for fine root biomass, fine root necromass and fine root production and examined the potential to improve model performance by recalibrating model parameters. The selection of the input variables was based on the Bayes information criterion, which strongly penalizes the number of predictor variables than the Akaike information criterion (AIC; Akaike, 1974; Burnham & Anderson, 2004). The tested input variables were latitude, longitude, year of sampling (if data for several years were reported, we used the mean), age of forest stand, T, P, LAI, and sampling depth (see previous sections). We analysed the effect of tree species composition by including dummy variables on dominant species type (conifers were assigned ‘0’, broadleaves ‘1’) and the presence of multiple species within the forest stand (monospecific stands were assigned ‘0’, mixed stands ‘1’). We also used dummy variables for the sampling method. Our dummy variable grouping for fine root biomass and necromass considered coring, trenches and monoliths, and for fine root production ingrowth cores/bags, sequential coring and minirhizotrons.

For all three fine root metrics, we used 10‐fold cross‐validation, a commonly used method to evaluate the model performance (McLachlan, Do, & Ambroise, 2004). The observations were split into 10 equal‐sized subsamples, one subsample was retained and the rest were used as training data for the model. This procedure was repeated 10 times and the average performance indicators were computed.

Table 1 provides an overview of the available fine root data and the represented environmental conditions and forest structure, separated by our three bioregions (Figure 1). We calculated the stand density index after Reineke (1933) as a measure of tree competition within the forest stand (Stand density index = stem number × (diameter/25)^1.605^), which considers that larger trees require more space.

**Table 1:** Summary statistics (mean, minimum and maximum in parentheses) for all fine root observations. For stem number, we used the median instead of the mean to accommodate the skewness. Stand density index was calculated after Reineke (1933)

	All	North	Central	South
Plots, *n*	454	126	309	19
Elevation (m)	287.3 (3.0–1,617.0)	125.1 (3.0–430.0)	336.5 (21.9–1,617.0)	656.6 (12.0–1,428.0)
Mean annual temperature (°C)	6.9 (−2.2–15.3)	4.0 (−2.2–7.6)	7.9 (3.1–13.6)	12.0 (5.0–15.3)
Mean annual precipitation (mm)	739 (457–1,521)	635 (457–858)	783 (518–1,521)	706 (469–1,197)
LAI (m^2^/m^2^)	4.4 (0.8–6.7)	4.3 (1.2–6.3)	4.4 (1.3–6.7)	3.6 (0.8–6.4)
NPP (g C m^−2^ year^−1^)	529 (282–971)	437 (282–613)	560 (313–971)	590 (298–851)
Basal area (m^2^/ha)	26.9 (0.3–63.0)	22.0 (0.3–54.0)	29.3 (0.3–63.0)	22.8 (21.0–26.6)
Stem number (ha^−1^)	673 (95–141,000)	1,311 (199–100,000)	460 (102–141,000)	759 (95–18,500)
Age (years)	75.9 (2–250)	60.2 (4–220)	82.9 (2–250)	64.7 (10–200)
Tree diameter (cm)	25.7 (0.8–103.7)	17.8 (1.3–36.7)	29.2 (0.8–103.7)	20.1 (10.4–28.0)
Stand density index	653 (17–4,833)	666 (17–4,717)	658 (20–4,833)	402 (50–685)
Foliage biomass (g/m^2^)	547 (7–2,736)	600 (7–2,495)	529 (8–2,736)	387 (55–988)

## 3 RESULTS

### 3.1 Fine root biomass, necromass and production in European forests

**Figure 2:**
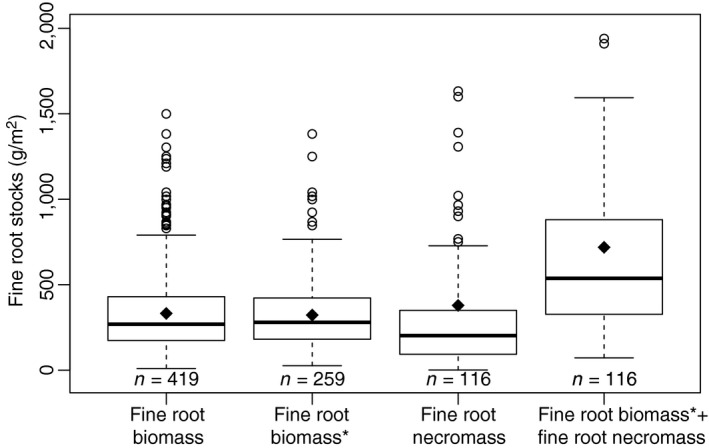
Fine root biomass and necromass. Here ‘biomass’ includes all reported fine root biomass observations (covering also observations where it is ambiguous whether dead roots were also assessed), while ‘biomass*’ are the biomass observations only covering living fine roots. ‘necromass’ represents the reported fine root necromass and finally ‘biomass* + necromass’ is the sum of fine root biomass and necromass for plots that reported both stocks. The boxes represent the median and the 25th and 75th percentile, the diamonds the arithmetic means, the whiskers extend to 1.5 of the interquartile range, values outside this range are indicated by circles. The number of observations (*n = ...*) are given below the bars

Our updated database had 419 observations of fine root biomass for European forests. The mean fine root biomass was 332 g/m^2^ (Figure 2, Table 2), which is a value comparable to the previous studies of temperate and boreal forests (Finér et al., 2011a) but smaller than previous estimates for boreal forests (Yuan & Chen, 2010). In 259 observations, it was explicitly stated that dead roots (necromass) were removed (average fine root biomass* 323 g/m^2^), and these studies represent only living fine root biomass. The average fine root necromass was 379 g/m^2^ (*n* = 116), and the total fine root biomass, including living and dead fine roots, was 722 g/m^2^ (Figure 2). Since the distribution of fine root necromass data were strongly skewed as evident in Figure 2, the fine root total mass was also skewed.

**Table 2:** Summary data of fine root observations by region and dominant species type. Shown is the sampling depth as reported in the literature, fine root biomass, necromass, production and carbon fraction of fine roots, followed by the respective number of observations (*n*). Data shown are mean ± standard deviation

	All	North		Central		South	
		Conifers	Broadleaves	Conifers	Broadleaves	Conifers	Broadleaves
Sampling depth (cm)	38.8 ± 24.8	32.8 ± 13.3	44.3 ± 13.1	44.7 ± 32.3	39.1 ± 25.0	15 ± 8.7	38.8 ± 10.5
*n*	450	98	23	125	176	3	12
Fine root biomass (g/m^2^)	332 ± 234	284 ± 152	199 ± 101	329 ± 223	385 ± 258	618 ± 628	248 ± 240
*n*	419	98	23	127	176	5	12
Fine root necromass (g/m^2^)	379 ± 569	255 ± 332	94 ± 91	288 ± 272	685 ± 870	—	97 ± 52
*n*	116	22	8	42	36	0	5
Fine root production (g m^−2^ year^−1^)	250 ± 215	296 ± 275	200 ± 101	173 ± 184	284 ± 198	—	303 ± 214
*n*	99	32	8	25	33	0	9
Carbon fraction (%)	48.4 ± 2.3	47.3 ± 0.9	49.4 ± 1.9	47.5 ± 2.1	47.6 ± 2.5	—	53.1 ± 0.6
*n*	44	11	13	10	6	0	3

Table 2 provides a summary data of the collated database on fine roots. Mean and standard deviation of fine root biomass, fine root necromass, fine root production and carbon fraction are shown by regions (Figure 1) and by tree species type (Table 2). The number of observations sometimes did not add up in Tables 1 and 2 due to missing information on species type and/or sampling depth in some references.

**Figure 3:**
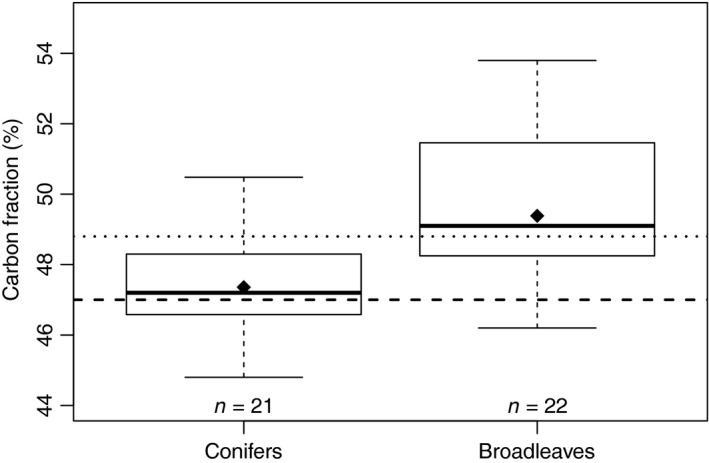
Observed carbon fraction in fine roots separated into conifers and broadleaves and compared to literature estimates. The dashed horizontal line represents the default carbon fraction of 47% for carbon reportings (Intergovernmental Panel on Climate Change, 2006) and the dotted line the value reported by Jackson et al. (1997) of 48.8%. For description of box and whiskers plots, see Figure 2

Available information on the C fraction in fine roots in European forests was rather scarce (*n* = 44, Table 2). The carbon fraction of broadleaves fine roots was larger than that of conifers (Figure 3). The difference in the mean carbon fraction between broadleaves and conifers was about +1.5%, which was statistically significant at *p* = .052 (difference in variance *p* = .957). However, there was substantial variation in the reported carbon fraction at European scale (conifers minimum 44.8%, maximum 55.0%, standard deviation 2.4%, broadleaves minimum 44.8%, maximum 53.8% and standard deviation 2.4%). In Figure 3, we show the observed carbon fractions in comparison with two literature‐based carbon fraction estimates, 47% suggested by the IPCC for Greenhouse Gas reporting (Intergovernmental Panel on Climate Change, 2006) and 48.8% reported for global forests by Jackson et al. (1997).

### 3.2 Evaluation of estimation models

We compared the outputs of 19 independent published models using data compiled by this study (9 models for fine root biomass, 10 models for fine root production, Table 3). For this comparison, we used observed values for reported sampling depth (Table 2) and extrapolated values using Eq. 1 and parameters from literature (Gale & Grigal, 1987; Jackson et al., 1997). We used three error estimators (RMSE, MAE and bias) and coefficients of determination. Root‐mean‐square error places more weight on extreme values, while MAE is easier to interpret and each observations has the same weight (Pontius, Thontteh, & Chen, 2008). We also show in Table 3, mean error and coefficient of determination of all models, for example, to evaluate the model envelope or to check the effect of extrapolation to maximum rooting depth on the combined model performance. We removed the missing model output values from analysis (e.g. due to missing climate or biomass input data) in Table 3. All results of error analysis are shown in Table S2.

**Table 3:** Results of error analysis of all models versus fine root biomass (a) and fine root production (b) after removing missing values to keep the number of observations (*n*) constant. We show the root‐mean‐square error (RMSE), mean absolute error (MAE), bias and coefficient of determination (*R* ^2^; Willmott & Matsuura, 2006). On the left section of the table, we compare model outputs with published data, on the right side, we evaluate the data as extrapolated to maximum rooting depth (Jackson et al., 1997)

Models		versus observed fine root biomass					versus extrapolated fine root biomass			
Equations	Description	*n*	RMSE	MAE	Bias	*R* ^2^	RMSE	MAE	Bias	*R* ^2^
*(a) Model predictions versus observed or extrapolated fine root biomass*										
B1	LAI MOD17	139	402	343	236	.001	463	335	63	.005
B2	LAI White	139	842	670	603	.009	809	616	430	.017
B3	LAI Pietsch	139	344	246	−26	.010	514	345	−199	.018
B4	Yuan	139	330	261	121	.042	422	276	−52	.054
B5	Liski	139	303	214	−73	.067	483	310	−246	.056
B6	Harkonen	139	301	215	−49	.086	458	311	−222	.110
B7	Foliage biomass	139	573	390	229	.013	508	361	63	.138
B8	Foliage White	139	294	205	−53	.076	443	297	−226	.165
B9	Foliage Pietsch	139	361	256	−247	.086	576	424	−420	.125
Model mean		—	417	311	82	.043	520	364	−90	.077

Models		versus observed fine root production					versus extrapolated fine root production			
Equations	Description	*n*	RMSE	MAE	Bias	*R* ^2^	RMSE	MAE	Bias	*R* ^2^
*(b) Model predictions versus observed or extrapolated fine root production*										
P1	Malhi NPP	77	183	153	91	.005	542	211	−78	.013
P2	LAI MOD17	77	471	425	415	.001	630	426	247	.005
P3	LAI White	77	282	253	229	.002	552	285	60	.001
P4	LAI Pietsch	77	170	118	−86	.001	598	263	−255	.000
P5	Liu total T + P	77	184	159	119	.063	537	208	−50	.021
P6	Liu total T	77	188	165	122	.035	537	215	−48	.021
P7	Liu leaf T + P	77	141	112	21	.063	556	202	−149	.021
P8	Liu leaf T	77	144	115	24	.035	555	201	−145	.021
P9	Liu leaf T + P WC	77	140	108	5	.064	560	207	−164	.029
P10	Liu leaf T WC	77	141	109	9	.045	558	205	−161	.034
Model mean		—	204	172	95	.031	562	242	−74	.017

**Figure 4:**
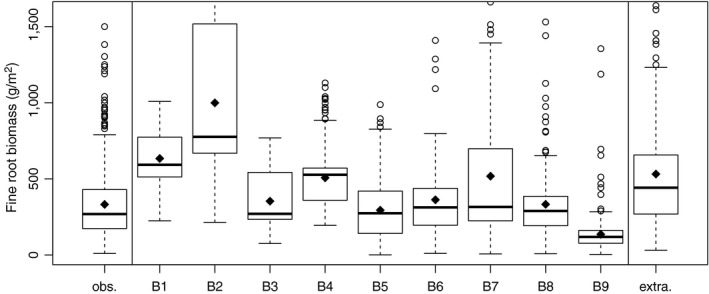
Fine root biomass observed (obs.) and calculated with the nine models. From left to right, we show the results based on leaf area index and global conversion factors from the MOD17 algorithm (model B1; Running & Zhao, 2015), model B2 using North American coefficients (White et al., 2000) as well Model B3 using European factors (Pietsch et al., 2005). Model B4 provides estimates assuming fine root biomass is 2% of stem biomass (Liski et al., 2002). Model B5 uses foliage biomass (Härkönen et al., 2011) and model B6 estimates fine root biomass based on latitude (Yuan et al., 2018). Model B7 assumes that fine root biomass is equivalent to foliage biomass and models B8 and B9 use foliage biomass and the fine root–leaf ratios of Pietsch et al. (2005) and White et al. (2000), respectively for estimating fine root biomass. Finally, we show extrapolated fine root biomass for entire rooting depth (extra). The number of observations was 139 as in Table 3. For description of box and whisker plots, see Figure 2

**Figure 5:**
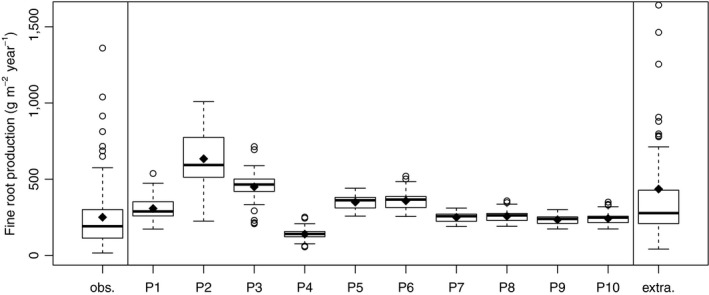
Fine root production observed (obs.) and calculated with the 10 different models. From left to right, we show the results of model P1, which assumes that fine root production is a constant fraction of net primary production (Malhi et al., 2011). Model P2 shows estimates based on leaf area index and global and turnover conversion factors (Running & Zhao, 2015), while Model 3 uses parameters from North America (White et al., 2000). Model P4 combines leaf area index with Central European coefficients (Pietsch et al., 2005). Models P5 to P10 assume fine root production is equivalent to above‐ground litterfall (Raich & Nadelhoffer, 1989) and models P5 and P6 estimate fine root production using the total litterfall function of Liu et al. (2004), while models P7 to P10 use the leaf litterfall function from the same reference. Models P5, P7 and P9 are based on temperature and precipitation and P6, P8 and P10 estimate litterfall only with temperature. Finally, we also show fine root production extrapolated for entire rooting depth (extra.). The number of observations is 77 as used for Table 3. For description of box and whiskers plots see Figure 2

We extended our model evaluation (Figure 4) by comparing outputs of fine root biomass models with published field data, and as extrapolated to entire rooting depth (Jackson et al., 1997) using boxplots. We did a similar comparison also for fine root production models (Figure 5). Boxplots enable assessment of variation and extreme values. We again excluded observations with missing model outputs.

**Figure 6:**
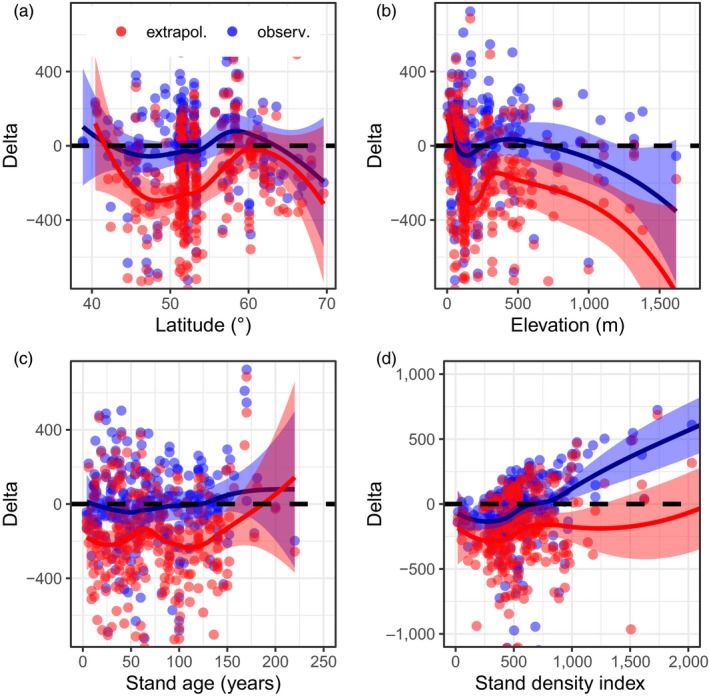
Evaluation of model B8 (‘Foliage White’) output versus selected gradients. We show the residuals (Delta) using original observations (‘observ.’ in blue) and extrapolated values (‘extrapol.’ in red). Plot a shows residuals by latitude, plot b by elevation, plot c by mean stand age and plot d by Stand density index. In addition to single values, we show the smoothed mean and its confidence band. For similar plots on other model outputs, see the Supporting Information [Colour figure can be viewed at http://wileyonlinelibrary.com]

**Figure 7:**
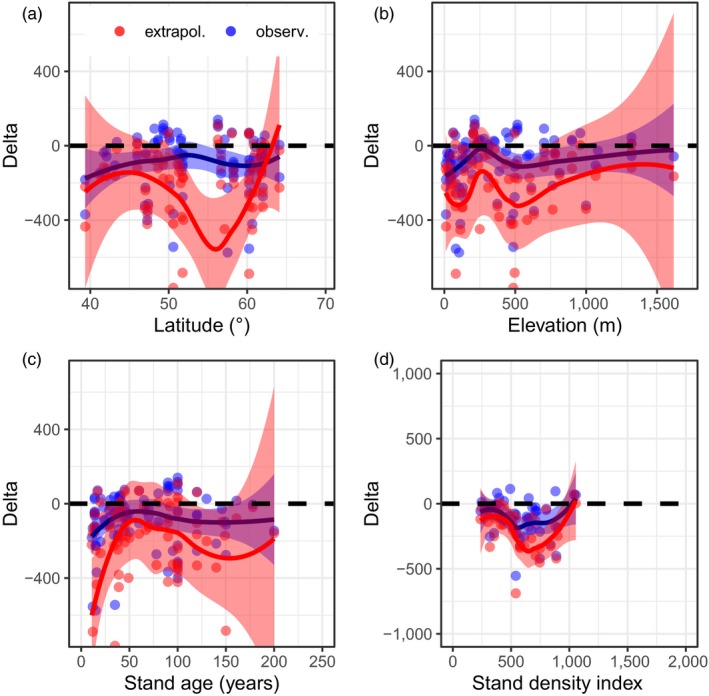
Evaluating model output P4 (‘Pietsch LAI’) versus selected gradients. For details see Figure 6 [Colour figure can be viewed at http://www.wileyonlinelibrary.com]

Overall, models only weakly estimated fine root biomass and production. The average coefficient of determination was 0.043 for fine root biomass, and 0.031 for fine root production (Table 3). Models based on biomass (B5, B6 and B8), on LAI (P4, B3), or assuming fine root biomass is equivalent to foliage mass (B7), better captured variation and exhibited less error when compared to observations, than models using climate (P5–P6) and latitude (B4) as input variables (Figures 4 and 5, Table 3). In addition, Figures 4 and 5 indicate that some models produced more skewed outputs (e.g. models P2, B2, and B7 vs. B3–B6). Model outputs in general, agreed better with observed fine root biomass and fine root production than with values extrapolated to maximum rooting depth (Table 3). Plotting residuals against selected gradients (latitude, elevation, stand age and stand density, Figures S1–S19) provided confirmation. For fine root production, least error and bias were found for climate‐sensitive models of Liu et al. (2004; models P5–P10). We tested the models of Liu et al. (2004) with two input datasets, without improving model performance (models P7 and P8 vs. P9 and P10, Table 3). Several models had similar errors in output, but varying bias and coefficients of determination (for fine root biomass B5, B6, B8 and B9, for fine root production P4 and P7–P10). For two biomass‐based models (models B8 and P4), we show residuals (observations minus predictions) across gradients to demonstrate discontinuities in model predictions (Figures 6 and 7, other models are shown as Figures S1–S19). For instance, model B8 exhibits positive error/underestimation at a stand density index larger than 800 in Figure 6, while model P4 exhibits negative error/overestimation at elevations lower than 250 m in Figure 7.

Model outputs for both fine root biomass and fine root production in general matched better with observations than extrapolated data in terms of error and bias, despite some models having reduced bias and greater coefficient of determination compared to extrapolated data (Table 3). Most models also agreed better with observations than with extrapolated data along the four selected gradients (Figures 6 and 7; Figures S1–S19). While there was large variation in residuals, models B8 and P4 reproduced observed patterns in fine root biomass and production, in particular along latitudinal and stand age gradients.

Overall, some models could approximate fine root biomass or fine root production and capture large‐scale gradients, but failed to describe observed variation (models explained < 10% of observed variation). Curiously, comparing outputs of some models with extrapolated values increased coefficients of determination (Table 3). This suggests that other drivers of fine root biomass and fine root production were confounding model predictions. Refitting models, combining them and/or including additional input variables might provide more accurate estimates.

### 3.3 Sources of variation in fine root biomass, fine root necromass and fine root production

We explored sources of variation in fine root biomass and fine root production by fitting multiple linear models to test if recalibration could improve model performance. We also examined sources of variation in root necromass, since this represents a substantial share of fine root stocks (Figure 2), and no estimation models were available.

There was ~4‐fold more data for fine root biomass than for fine root necromass and fine root production (Table 4). After cross‐validation, our models produced coefficients of determination for both fine root biomass and fine root production (Table 4) which were more than threefold greater (>0.32) than that of the best performing published models (Table 3). Highly significant input variables (*p* < .001) for estimating fine root biomass were mean annual precipitation, sampling depth and the dummy variables for dominant tree species type and species mixing. The dummy variable for sampling method was not significant. For fine root production only sampling depth was highly significant (*p* < .001). Only the dummy tree species type had a significant effect (*p* = .016) on fine root necromass predictions.

**Table 4:** Result of analysis of 10 input variables (Age, Latitude, Longitude, Mean annual temperature, Mean annual precipitation, leaf area index (LAI), Sampling depth, Sampling year) and three dummy variables (Dummy method, Dummy dominant species type, Dummy mixing) using multiple linear models. *n* is the number of samples, *R* ^2^ coefficient of determination, root‐mean‐square error (RMSE) and mean absolute error (MAE) of 10‐fold cross‐validation. We only show the results for the best model based on an information criterion framework and the variable selection was based on Bayes information criterion by Akaike (1974). Shown are the results for fine root biomass (a), followed by fine root necromass (b) and fine root production (c)

Input variable	*n* = 373, *R* ^2^ = .319, RMSE = 186.4, MAE = 141.7			
	coef	coef *SE*	*t* value	*p* value
(a) Fine root biomass				
Intercept	−1.25E+03	2.68E+03	−0.468	.640
Age (year)	1.12E−01	2.20E−01	0.510	.610
Mean annual precipitation (mm)	1.39E−01	4.02E−02	3.471	<.001
LAI (m^2^/m^2^)	−1.33E+01	6.88E+00	−1.927	.055
Sampling depth (cm)	4.32E+00	3.86E−01	11.207	<.001
Sample year (year)	6.46E−01	1.34E+00	0.483	.629
Dummy mix	1.26E+02	3.06E+01	4.111	<.001
Dummy dominant species type	8.07E+01	2.29E+01	3.524	<.001

Input variable	*n* = 105, *R* ^2^ = .146, RMSE = 536.7, MAE = 372.2			
	coef	coef *SE*	*t* value	*p* value
(b) Fine root necromass				
Intercept	6.43E+02	3.90E+02	1.65	.102
Age (year)	8.44E−01	1.34E+00	0.628	.531
Mean annual temperature (°C)	1.56E+00	3.29E+01	0.047	.962
LAI (m^2^/m^2^)	−8.78E+01	6.23E+01	−1.41	.162
Dummy dominant species type	3.13E+02	1.26E+02	2.488	.015

Input variable	*n* = 86, *R* ^2^ = .338, RMSE = 142.3, MAE = 108.0			
	coef	coef *SE*	*t* value	*p* value
(c) Fine root production				
Intercept	1.12E+04	3.58E+03	3.114	.003
Age (year)	−6.14E−01	3.55E−01	−1.730	.088
Mean annual temperature (°C)	8.49E+00	7.41E+00	1.146	.255
LAI (m^2^/m^2^)	−2.26E+01	1.46E+01	−1.555	.124
Sampling depth (cm)	2.80E+00	6.69E−01	4.185	<.001
Sample year (year)	−5.49E+00	1.80E+00	−3.057	.003
Dummy mix	1.23E+02	4.54E+01	2.713	.008
Dummy dominant species type	1.05E+02	3.77E+01	2.788	.007

While we also tested if adding forest structure information as input variables (foliage biomass, stand density index) improved model performance, large amounts of missing data precluded a substantive analysis. Limiting model inputs to highly significant variables (mean annual precipitation, sampling depth, dummy dominant species type and dummy mix) shown in Table 4, reduced model performance for fine root biomass and produced an *R* ^2^ of .265. The same approach for fine root production (sample depth was the only highly significant input variable) reduced *R* ^2^ to .072.

## 4 DISCUSSION

### 4.1 Synthesizing available data

We compiled for Europe, a comprehensive and up‐to‐date database on fine root biomass, fine root necromass, fine root production and carbon fractions of fine roots. Average fine root biomass in Europe (332 g/m^2^) is similar to results obtained by a Eurasian study of 287 and 389 g/m^2^ for boreal forests and temperate forests respectively (Finér et al., 2011a). Mean values derived here for fine root biomass of conifers (284 g/m^2^) and for broadleaved species (199 g/m^2^) in northern Europe compare well with an earlier global review for boreal forests of 230 g/m^2^ (Jackson et al., 1997). For fine root production, our European average of 250 g m^−2^ year^−1^ is ~20% less than previous estimates of 311 g m^−2^ year^−1^ for boreal forests and 428 g m^−2^ year^−1^ for temperate forests of entire Eurasia (Finér et al., 2011b). Our average fine root production (250 g m^−2^ year^−1^) multiplied with mean C fraction (48.4%) results in a slightly smaller C flux (121 g C m^−2^ year^−1^) compared to C in above‐ground foliage litterfall of 134 g C m^−2^ year^−1^ (Neumann et al., 2018). Calculated product of fine root biomass and necromass (711 g/m^2^) and mean C fraction suggests fine root mass represents about 5% of total tree carbon (~7,300 g C m^−2^; Neumann, Moreno, Thurnher, et al., 2016) but turns over quickly.

### 4.2 Model evaluation and recalibration

We assessed models of fine root biomass and fine root production against independent field observations. All 19 tested models performed poorly, with coefficients of determination <0.1 and RMSE close to mean values. We can only speculate as to underlying reasons for this generally poor performance.

#### 4.2.1 Foliage biomass as a predictor of fine root biomass and fine root production

While our study suggests that foliage biomass can be a suitable proxy for fine root biomass and fine root production, both model parameters and input data remain key to accurate results. For fine root biomass, the best performing, in terms of error, was model B8 (MAE 205 g/m^2^, bias −53 g/m^2^, *R* ^2^ .076) which relies on values of foliage biomass and North American estimates of the ratio of fine root‐to‐foliage (FR:LEAF). Surprisingly, model B9 which uses FR:LEAF ratios collated for European forests, performed less well. The 50% greater FR:LEAF ratios for North America are a better basis for predicting patterns of fine root biomass in Europe, when foliage biomass is used as a model input. If European FR:LEAF ratios are used as inputs, then model B3 and LAI obtained from remote sensing provide the most accurate predictions of fine root biomass. It is worth noting that foliage biomass alone is not a good predictor of fine root biomass, as seen from the output of model B7. Model B7 had poorer performance than models which used species type‐specific FR:LEAF ratios as input data (models B6, B8 and B9).

For fine root production, the best performing model was P4 (MAE 118 g m^−2^ year^−1^). Model P4 uses LAI obtained from remote sensing and FR:LEAF ratio values compiled for Central Europe (Pietsch et al., 2005). Model P4 used the same LAI as used for models P2 and P3, but different model parameters. The Biome BGC parameters of Central Europe used for P4 (Pietsch et al., 2005) are more suitable than the North American parametrization (White et al., 2000) used in P2, or the global MOD17 algorithm used in P3.

While somewhat contradictory, no single set of model inputs (Pietsch et al., 2005; White et al., 2000) resulted in best estimates of both fine root biomass and fine root production. More accurate and comprehensive analysis of forest structure and foliage biomass would assist in developing predictive functions for root parameters, and for below‐ground carbon stocks and turnover (sensu Neumann, Moreno, Mues, et al., 2016).

#### 4.2.2 Use of remote sensing and climate data in predicting fine root parameters

For large regions of the world, there remain few direct measurements of foliage biomass or LAI (FAO, 2015; Pan et al., 2011). In contrast, remote sensing data are available worldwide and can provide estimates of LAI that can be converted into foliage biomass (Yan et al., 2016; Yang et al., 2006). As discussed above, model P4 used LAI inputs and provided the best estimates of fine root production. Remotely sensed LAI combined with appropriate parameters described fine root production with similar accuracy to climate‐sensitive models, which assume proportionality between foliage litterfall and fine root production (models P7–P10). Models P7–P10 have reduced bias and greater coefficients of determination than P4. Climate data are also available worldwide (Hijmans et al., 2005) and a potential advantage of models based on climate (P7–P10), or models based on latitude, is their wider applicability. Models that used remotely sensed NPP and LAI had poorer coverage than models based on climate. In addition, a considerable proportion of available fine root data are derived from fragmented forests (frequent in Central Europe) in urban or agricultural lands. Remote sensing pixels for these sites captures dominant non‐forest land cover, and derived estimates of LAI cannot be reliably used to estimate fine roots. Nonetheless, when combined with remotely sensed foliage estimates, models based on Central European Biome BGC parameters produced estimates of fine root biomass and fine root production with low bias and error. We have assessed here simple models and related input variables. We used a global LAI dataset at 1‐km resolution (Yang et al., 2006) and we did not consider regional variations in inputs, that is, the fine root biomass and production of all conifer forests were calculated with the same parameters. Foliage biomass estimates were also derived from a set of simple allometric biomass functions (Neumann, Moreno, Mues, et al., 2016) not able to capture local variation in tree allometry. Better model performance could be expected if there were available, local biomass functions, higher resolution LAI maps and regional conversion factors.

#### 4.2.3 Potential of recalibrating multi‐regression models

Our model validations suggest that both remotely sensed and foliage biomass estimates are correlated with fine root biomass and fine root production, and these correlations are stronger than that with latitude, stem biomass or NPP. Not surprisingly, multiple‐regression models better explained observed fine root biomass and fine root production than evaluated models.

Precipitation and temperature were positively correlated with all fine root metrics in our fitted models, while LAI was negatively correlated. Hence, after taking into account differences in climate, LAI was inversely related to fine root biomass. Our study provides fresh evidence that ratios of leaves to fine roots are not constant (Chen et al., 2018; Malhi et al., 2011) and we caution against use of models that make such an assumption.

When we recalibrated models, we could reduce RMS error by 42% for fine root biomass (−22% for fine root production), and MAE by 45% (−16% for fine root production), compared to the best published model (B4 for fine root biomass, P4 for fine root production). Coefficients of determination of recalibrated models were all >0.3—three to fourfold greater than those of published models. Nonetheless, there is ample scope to improve understanding of the processes and drivers of fine root dynamics.

#### 4.2.4 Species type and mixture

Both model evaluation and recalibration highlight that fine root biomass and fine root production are related to forest stand conditions, including tree stem dimensions and tree species composition. Tree species composition (dominance and mixing) appeared to be a key driver for fine root stocks and fluxes, as suggested by earlier research (e.g. Brassard, Chen, Bergeron, & Paré, 2011; Finér et al., 2011b; Schmid & Kazda, 2002). Comparisons are made difficult by the differing geographic distributions, and prevailing climates, of broadleaved species and conifers in Europe (Forest Europe, 2015). Even after accounting for differences in climate, stand age and sampling depth, broadleaved as well as mixed stands still show greater fine root biomass, necromass and production.

### 4.3 Increasing accuracy of fine root assessments

Here we address future research priorities for the following: (a) depth distribution of fine roots, (b) estimation of necromass, (c) temporal variation in fine root biomass and fine root production and (d) carbon fraction of fine roots.

#### 4.3.1 Rooting depth

Extrapolating to entire rooting depths, fine root data as derived from the most frequently used sampling protocols, remains a difficult task. Even so, based on results shown here, we conclude that current estimates of fine root biomass and fine root production are most likely conservative. Simple extrapolation of observed fine roots to maximum rooting depth, directly by using the concept of Gale and Grigal (1987) and Jackson et al. (1997), or indirectly by excluding studies with a shallow sample depths (i.e. Wang et al., 2018; Yuan et al., 2018), will lead to erroneous estimates. Estimates of maximum rooting depth (sensu Jackson et al., 1997; Schenk & Jackson, 2002) are inevitably based on few observations and do not necessarily provide an unbiased picture of average rooting conditions for forests worldwide. Extrapolating fine root biomass and production seems most likely to provide realistic estimates only for sites with good soil conditions and deep rooting profiles (Kirfel, Heinze, Hertel, & Leuschner, 2019). As we have shown, extrapolation leads to greater uncertainty in model predictions of root parameters. Most reports fail to identify how sample depth relates to maximum rooting depth. Gridded root zone information may help to understand this relationship (Fan et al., 2013, 2017) since maximum rooting depth varies with not only mean annual temperature, mean annual precipitation, but also with soil hardness, groundwater level and soil organic matter.

#### 4.3.2 Necromass

The mass of living fine roots represents only about 45% of overall fine root mass (when necromass is considered), and in turn only a portion of the C stock in fine roots (Figure 2). Our results stress that necromass—accounting for more than half of fine root C—must be a priority in future efforts to quantify soil C stocks for Europe (see also Leuschner & Hertel, 2003; Wang et al., 2018). While there are major technical/economic difficulties in separating living roots from dead roots and soil particles, ignoring fine root necromass results in an incomplete picture of C stocks in forests. Our results reinforce the importance of fine root necromass generally to C stocks across Europe's forest ecosystems, which may become paramount in cold boreal forests where decomposition is slow (Zhang, Hui, Luo, & Zhou, 2008).

#### 4.3.3 Temporal variation

Fine root growth and mortality is not constant over time (Kummerow, Kummerow, & Trabaud, 1990; Montagnoli et al., 2018; Zhiyanski, 2014). With the exception of minirhizotrons (Leppälammi‐Kujansuu et al., 2014; Majdi, 2001), most methods only assess fine roots at selected points in time. Measurement periods may also be restricted by accessibility or frozen soil conditions and not all information in our database necessarily represents ‘average annual conditions’. For example, Brunner et al. (2013) demonstrated that bias in the timing of sampling will bias estimates of fine root biomass and probably also on those of necromass. Similarly, fine root biomass data for single years will most likely exhibit greater variability than fine root biomass averaged over multiple years. For C reporting, stocks averaged over entire years are probably most meaningful.

#### 4.3.4 Carbon fraction

Based on 44 observations of C concentrations in fine roots of European forests, we estimated a mean C fraction of 48.4%. Our study thus that the default carbon fraction for forest biomass suggested by the IPCC for Greenhouse Gas reporting of 47% (Intergovernmental Panel on Climate Change, 2006) citing McGroddy, Daufresne, and Hedin (2004) is somewhat conservative. Our estimate is roughly the midpoint of C fractions for global forests reported earlier by Jackson et al. (1997) of 48.8% (*n* = 4), and that reported by Thomas and Martin (2012) of 48.0% (*n* = 19). It is remarkable that we have C fraction data for just 30 sites across Europe, given many studies report carbon fraction by tree species for mixed forests. We strongly advocate that scientists make accessible their highly valuable data on C fractions, which will help us to better quantify C stocks and fluxes (particular important would be data for South Europe and for necromass). The scaling‐up of only small differences in C fraction has large implications for estimates of C storage. As an example, the forest area in our study region is ~182 million hectares (Forest Europe, 2015). If we calculate the product of forest area, average fine root biomass (332 g/m^2^), and C fraction suggested by IPCC of 47% (instead of our estimated value of 48.4%), then we reduce the estimate of C stocks in fine roots by 8.5 Mt C, equal almost to a 5% of annual change in forest C stocks in Europe (Forest Europe, 2015). This simple estimation neglects regional differences in fine roots carbon fraction and does not consider effects of root diameter and root order on carbon fractions (Pregitzer et al., 2002). However, it demonstrates the importance of fine roots and their carbon content for global carbon cycle and efforts to mitigate climate change.

## 5 CONCLUSIONS

Information presented here for European forests can also be used for selecting models for estimating fine root biomass and production in similar forest ecosystems elsewhere. Our newly developed models may be used for ongoing estimation of fine root parameters in Europe. We highlight that accurate fine root carbon information that can be leveraged via ongoing efforts of root scientists worldwide. Using observation‐based estimates for carbon assessments will likely lead to fine root assessments that are not only robust, but also ‘measurable’, ‘reportable’ and ‘verifiable’ (Winkler, 2008). The introduced database, if kept updated and complemented, may help to further advance our understanding of fine roots and their dynamics.

## AUTHORS' CONTRIBUTIONS

M.N. conceived the idea of this study and performed the analysis and visualizations; M.N., D.L.G., Y.H. and L.F. contributed to compiling the database and developed the methodology; M.N., D.L.G., Y.H. and L.F. discussed and revised the drafts of the manuscript written by M.N.

## Data Availability

The data used in this study are publicly available via: https://doi.org/10.6084/m9.figshare.7676219.v1 (Neumann, Godbold, Hirano, & Finer, 2019).
