# Supplementary material for: Improving models of fine root carbon stocks and fluxes in European forests
Source: J Ecol. 2020 Jan 10;108(2):496–514. doi: 10.1111/1365-2745.13328 (PMC7065197; doi:10.1111/1365-2745.13328)
Supplement: Supplementary file 1 [file JEC-108-496-s001.docx]

Supporting Information

**Improving models of fine root carbon stocks and fluxes in European forests**

Mathias Neumann ^1*^, Douglas L. Godbold ^2,3^, Yasuhiro Hirano ^4^, Leena Finer ^5^

* corresponding author, mathias.neumann@boku.ac.at

^1^ Institute of Silviculture, University of Natural Resources and Life Sciences, Peter-Jordan-Str. 82, 1190 Vienna, Austria

^2^ Institute of Forest Ecology, University of Natural Resources and Life Sciences, Peter-Jordan-Str. 82, 1190 Vienna, Austria

^3^ Global Change Research Centre, Academy of Sciences of the Czech Republic, České Budějovice 37005, Czech Republic

^4^ Graduate School of Environmental Studies, Nagoya University, Nagoya 464-8601, Japan

^5^ Natural Resources Institute Finland, Yliopistokatu 6, FI-80100 Joensuu, Finland

Table S1: Parameters for converting Leaf Area Index into fine root biomass (models B1–B3, B8 and B9) and fine root production (models P2–P4). We provide here parameters for the most important MODIS land cover types containing more than 10% forests. Land cover types containing below 10% forests involve croplands, grasslands, shrublands, barren land and urban area.

| Land cover type |  | Specific Leaf Area [m²kgC^-1^] | | | Turnover rate [year^-1^] | | | Fine roots to leaf ratio [ ] | | |
| --- | --- | --- | --- | --- | --- | --- | --- | --- | --- | --- |
|  | Name | MOD17 | White 2000 | Pietsch 2005 | MOD17 | White 2000 | Pietsch 2005 | MOD17 | White 2000 | Pietsch 2005 |
| 1 | Evergreen needle forests | 15.0 | 8.2 | 10.2 | 1 | 0.26 | 0.195 | 1.2 | 1.4 | 0.622 |
| 2 | Evergreen broadleaf forests | 26.9 | 32.0 | 41.3 | 1 | 1 | 1 | 1.1 | 1.2 | 0.545 |
| 3 | Deciduous needle forests | 16.9 | 8.2 | 20.3 | 1 | 1 | 1 | 1.1 | 1.4 | 1.000 |
| 4 | Deciduous broadleaf forests | 24.7 | 32.0 | 41.5 | 1 | 1 | 1 | 1.1 | 1.2 | 0.545 |
| 5 | Mixed forests | 22.6 | 20.1 | 25.9 | 1 | 0.63 | 0.598 | 1.1 | 1.3 | 0.584 |
| 8 | Woody Savannahs | 28.8 | 20.1 | 25.9 | 1 | 1 | 1 | 1.8 | 1.2 | 0.545 |
| 9 | Savannahs | 28.9 | 20.1 | 25.9 | 1 | 1 | 1 | 1.8 | 1.2 | 0.545 |

Table S2: Error analysis of all tested models versus fine root biomass (top) and fine root production (bottom), while Table 3 excluded observations with missing model output. We show root mean square error (rmse), mean absolute error (mae), bias and coefficient of determination (R^2^) (Willmott & Matsuura, 2006). On the left section of the table we compare model outputs with observations, on the right side we evaluate the extrapolated observations using maximum rooting depth (Jackson et al., 1997). The number of samples is indicated by *n* and changes due to missing model inputs for some field sites.

| Model prediction | |  | versus observed fine root biomass | | | | |  | vs. extrapolated fine root biomass | | | |
| --- | --- | --- | --- | --- | --- | --- | --- | --- | --- | --- | --- | --- |
| Eq. | description |  | *n* | rmse | mae | bias | R^2^ |  | rmse | mae | bias | R^2^ |
| B1 | LAI MOD17 |  | 290 | 408 | 349 | 263 | 0.000 |  | 485 | 346 | 61 | 0.001 |
| B2 | LAI White |  | 290 | 888 | 710 | 651 | 0.006 |  | 849 | 650 | 455 | 0.007 |
| B3 | LAI Pietsch |  | 290 | 331 | 243 | 7 | 0.006 |  | 531 | 353 | -191 | 0.007 |
| B4 | Yuan |  | 446 | 322 | 259 | 176 | 0.031 |  | 394 | 257 | -24 | 0.064 |
| B5 | Liski |  | 278 | 515 | 250 | 13 | 0.064 |  | 601 | 342 | -163 | 0.036 |
| B6 | Harkonen |  | 287 | 435 | 232 | 27 | 0.063 |  | 521 | 319 | -150 | 0.049 |
| B7 | Foliage biomass |  | 287 | 542 | 344 | 183 | 0.030 |  | 515 | 359 | 7 | 0.098 |
| B8 | Foliage White |  | 287 | 367 | 210 | -4 | 0.066 |  | 470 | 305 | -182 | 0.068 |
| B9 | Foliage Pietsch |  | 287 | 328 | 234 | -207 | 0.064 |  | 532 | 402 | -385 | 0.053 |
| **model mean** | |  | **-** | **416** | **302** | **111** | **0.039** |  | **517** | **363** | **-60** | **0.042** |
|  |  |  |  |  |  |  |  |  |  |  |  |  |
| Model prediction | |  | versus observed fine root production | | | | |  | vs. extrapolated fine root production | | | |
| Eq. | description |  | *n* | rmse | mae | bias | R^2^ |  | rmse | mae | bias | R^2^ |
| P1 | Malhi NPP |  | 99 | 201 | 164 | 75 | 0.000 |  | 605 | 249 | -125 | 0.001 |
| P2 | LAI MOD17 |  | 86 | 467 | 417 | 403 | 0.000 |  | 609 | 417 | 245 | 0.004 |
| P3 | LAI White |  | 86 | 283 | 253 | 218 | 0.005 |  | 531 | 281 | 60 | 0.002 |
| P4 | LAI Pietsch |  | 86 | 183 | 129 | -92 | 0.010 |  | 575 | 261 | -251 | 0.001 |
| P5 | Liu total T+P |  | 105 | 243 | 192 | 100 | 0.000 |  | 598 | 265 | -95 | 0.006 |
| P6 | Liu total T |  | 105 | 247 | 199 | 109 | 0.000 |  | 596 | 270 | -87 | 0.008 |
| P7 | Liu leaf T+P |  | 105 | 218 | 146 | 0 | 0.000 |  | 622 | 257 | -195 | 0.006 |
| P8 | Liu leaf T |  | 105 | 218 | 149 | 8 | 0.000 |  | 619 | 255 | -188 | 0.008 |
| P9 | Liu leaf T+P WC |  | 114 | 216 | 142 | -16 | 0.001 |  | 604 | 253 | -202 | 0.009 |
| P10 | Liu leaf T WC |  | 114 | 215 | 144 | -8 | 0.002 |  | 601 | 250 | -194 | 0.013 |
| **model mean** | |  | **-** | **249** | **193** | **80** | **0.002** |  | **596** | **276** | **-103** | **0.006** |


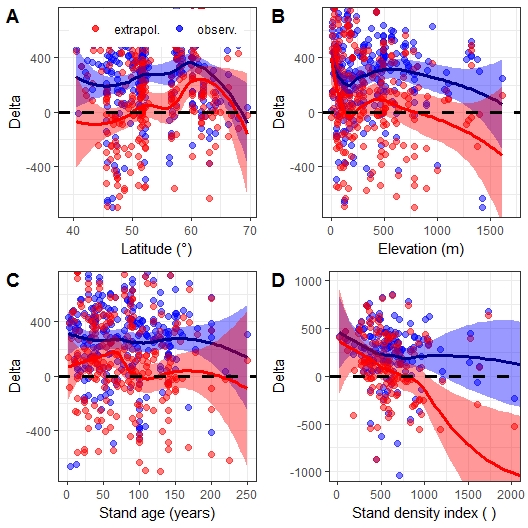


Figure S1: Evaluating model output B1 versus selected gradients. We show the residuals using original observations (“observ.” in blue) and extrapolated values (“Extrapol.” in red). Plot A shows residuals by latitude, plot B by elevation, plot C by mean stand age and plot D by Stand density index. In addition to single values we show the smoothed mean and its confidence band.


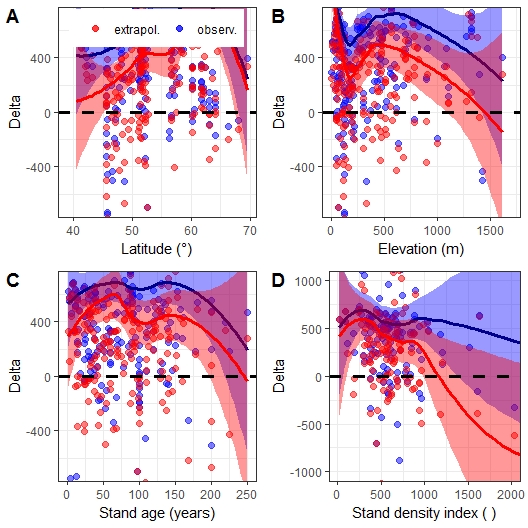


Figure S2: Evaluating model output B2 (for details see Fig. S1)


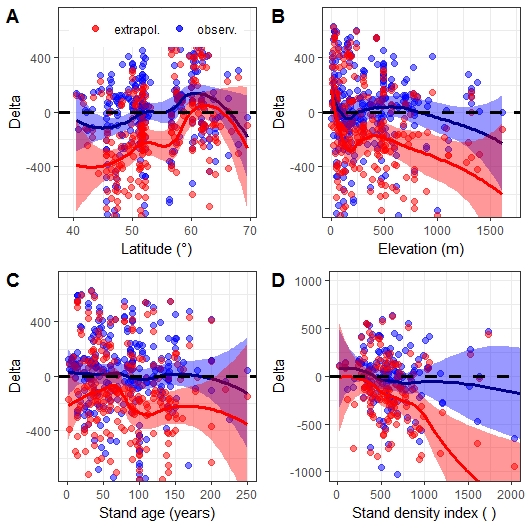


Figure S3: Evaluating model output B3 (for details see Fig. S1)


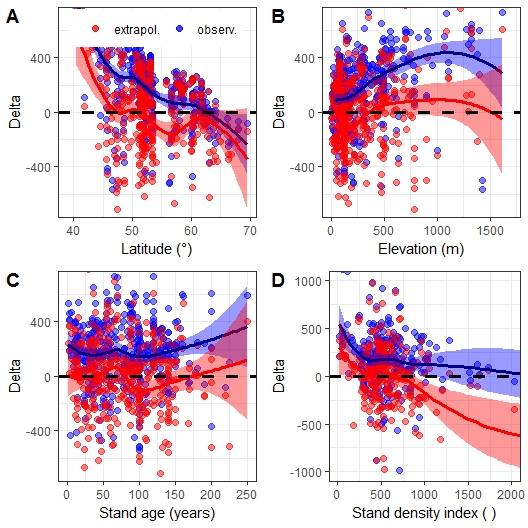


Figure S4: Evaluating model output B4 (for details see Fig. S1)


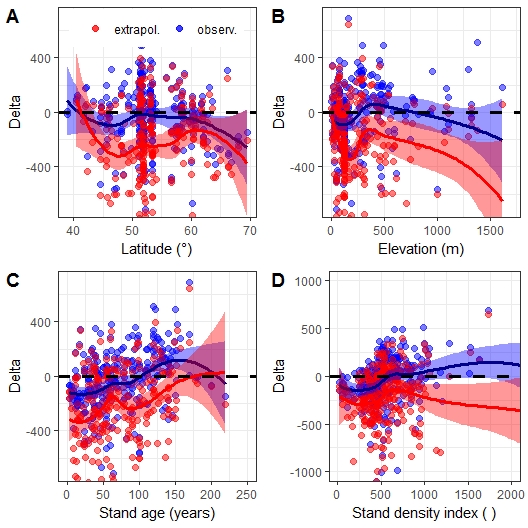


Figure S5: Evaluating model output B5 (for details see Fig. S1)


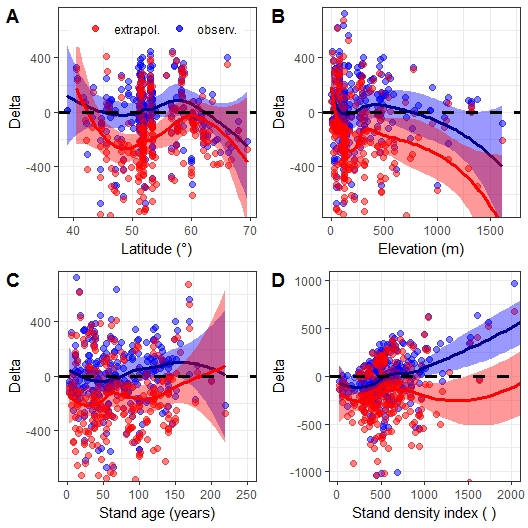


Figure S6: Evaluating model output B6 (for details see Fig. S1)


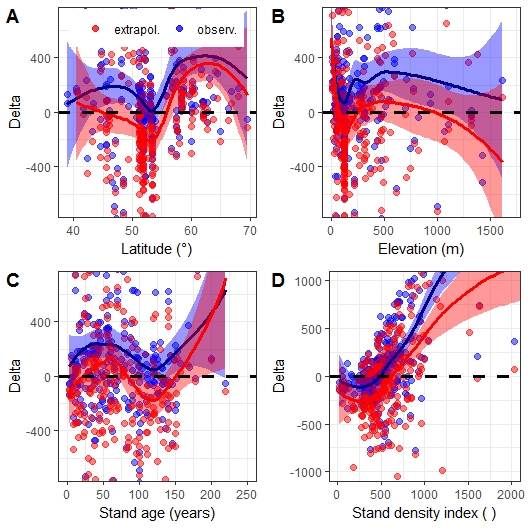


Figure S7: Evaluating model output B7 (for details see Fig. S1)


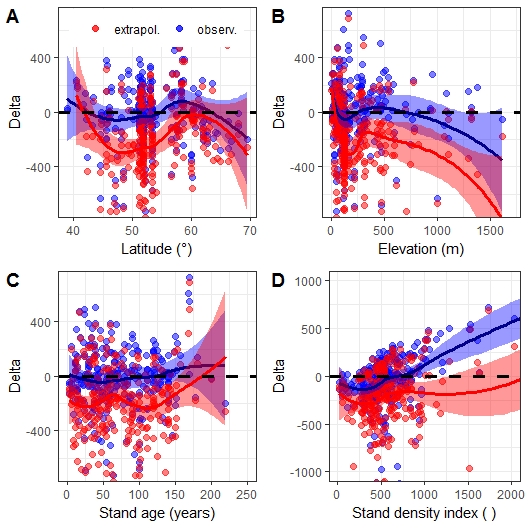


Figure S8: Evaluating model output B8 (for details see Fig. S1)


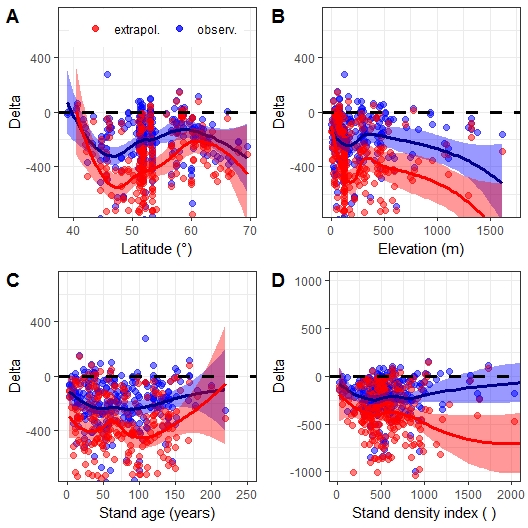


Figure S9: Evaluating model output B9 (for details see Fig. S1)


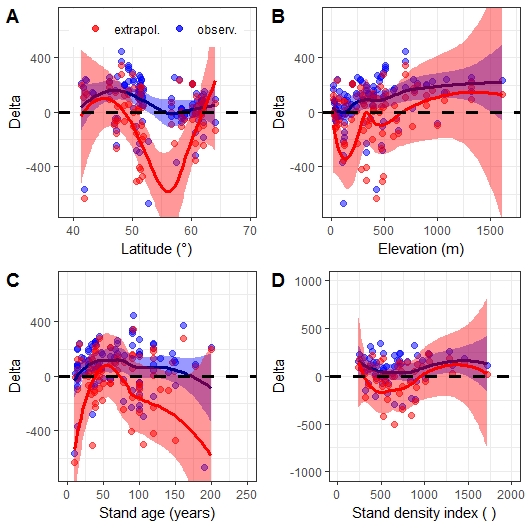


Figure S10: Evaluating model output P1 (for details see Fig. S1)


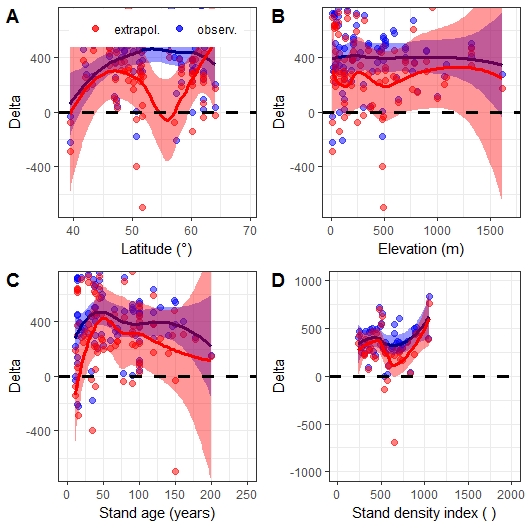


Figure S11: Evaluating model output P2 (for details see Fig. S1)


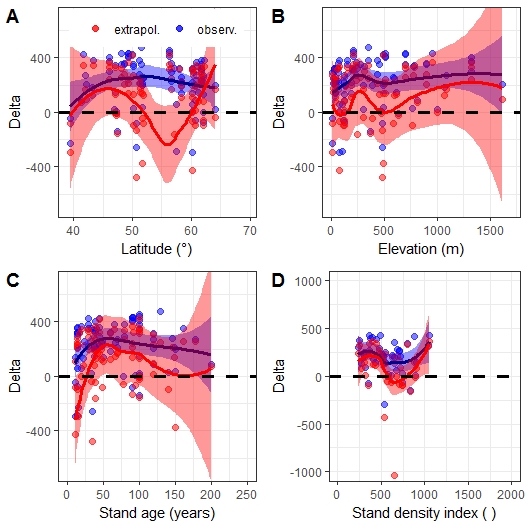


Figure S12: Evaluating model output P3 (for details see Fig. S1)


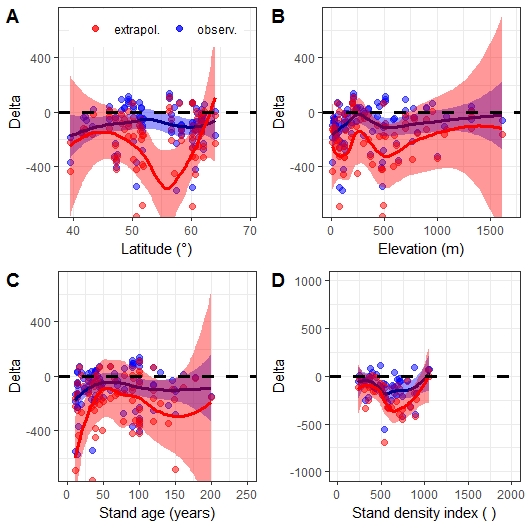


Figure S13: Evaluating model output P4 (for details see Fig. S1)


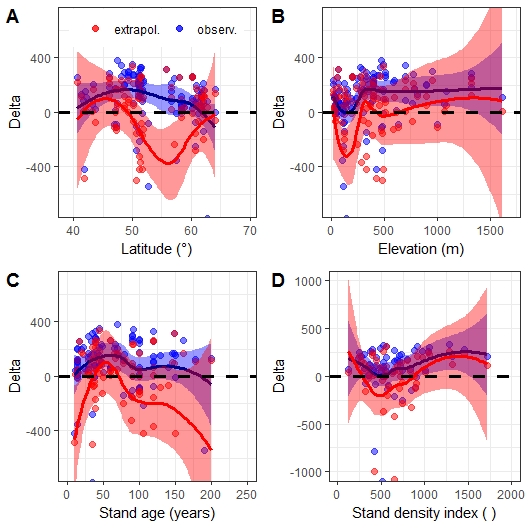


Figure S14: Evaluating model output P5 (for details see Fig. S1)


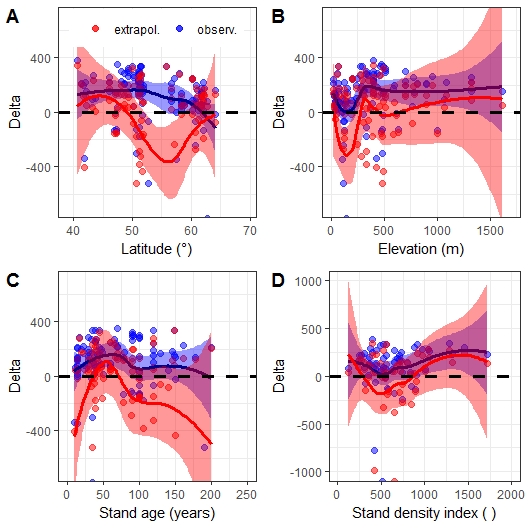


Figure S15: Evaluating model output P6 (for details see Fig. S1)


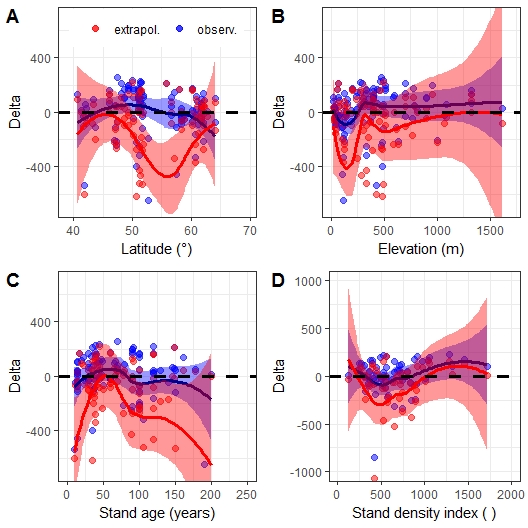


Figure S16: Evaluating model output P7 (for details see Fig. S1)


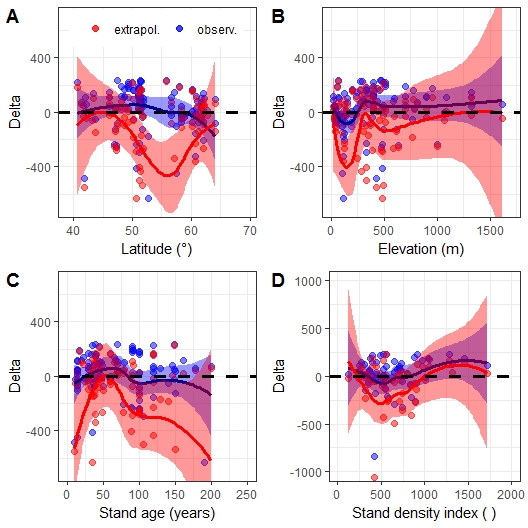


Figure S17: Evaluating model output P8 (for details see Fig. S1)


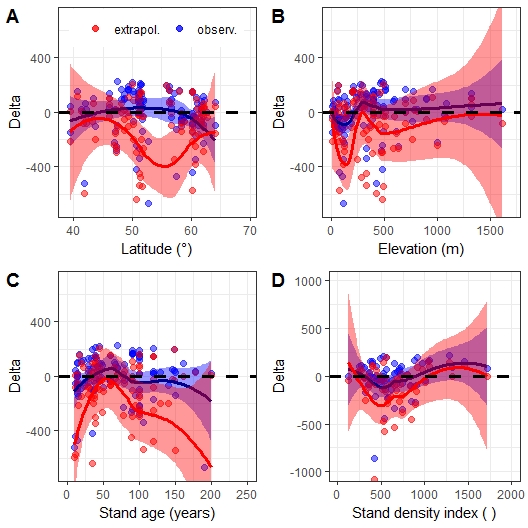


Figure S18: Evaluating model output P9 (for details see Fig. S1)


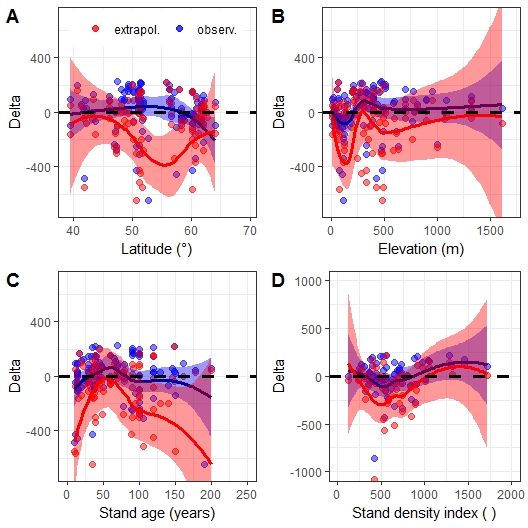


Figure S19: Evaluating model output P10 (for details see Fig. S1)


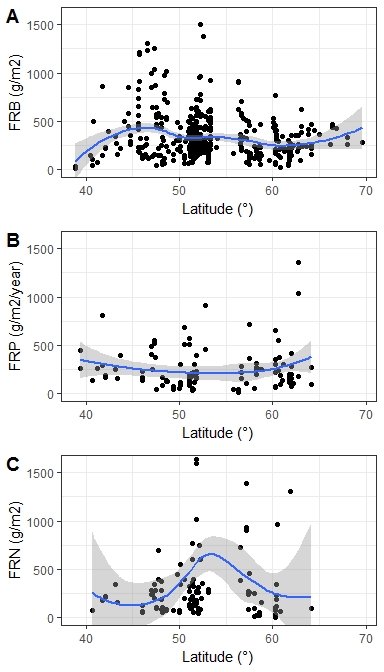


Figure S20: Fine root biomass (A), fine root production (B) and fine root necromass (C) versus latitude.
